# Sustainability: the role of the Surgical Center

**DOI:** 10.1590/1980-220X-REEUSP-2026-0177en

**Published:** 2026-06-01

**Authors:** Camila Quartim de Moraes Bruna

**Affiliations:** 1 Universidade de São Paulo, Escola de Enfermagem, Departamento de Enfermagem Médico-Cirúrgica, São Paulo, SP, Brazil.; 2 Universidade de São Paulo, Escola de Enfermagem, Comissão de Sustentabilidade, São Paulo, SP, Brazil.

## INTRODUCTION

The impact of human activities on the planet is well established, and there is much discussion surrounding it^(1,2,3)^. In contrast, less attention has been given to the impact of healthcare services on the planet^(4)^. It is recognized that healthcare services currently experience a vicious cycle, in which they generate significant emissions and impacts on the planet, which affect human health; consequently, people need to use healthcare services more, which generates further impact on the planet^(5)^.

It is believed today that if healthcare services were a country, they would rank fifth among the largest emitters of greenhouse gases^(6)^. Among hospital sectors, the Surgical Center, comprising the Operating Rooms (OR), Post-Anesthesia Care Unit (PACU), and Central Sterile Supply Department (CSSD), stands out as a major contributor to the carbon footprint^(7)^. Considering only the carbon footprint of SCs, estimates range from 28 kg to 505 kg of carbon dioxide equivalent (CO_2_e)^(8)^. Although emission levels vary widely depending on a number of factors, it is estimated that a single surgical procedure emits between 4 and 814 kgCO_2_e^(9,10)^.

This significant carbon footprint stems primarily from anesthetic gases and energy consumption^(7,10)^, but the large amount of waste generated, mainly from disposable materials^(8)^, also has a major impact. In the United States healthcare system alone, approximately 6 million tons of waste are generated annually^(11)^. Furthermore, a complicating factor is that a large portion of the waste generated in the OR is improperly sorted^(12)^.

The healthcare sector has become increasingly dependent on single-use materials, although these materials reflect an inherently unsustainable linear economic model, in which waste management creates a significant burden on public health in terms of environmental pollutants^(13)^. In this model, items are produced, used once, and then discarded.

Of the total waste generated in healthcare, 85% is considered general waste, comparable to household waste, while only 15% consists of infectious, toxic, carcinogenic, flammable, corrosive, reactive, explosive, or radioactive material^(14)^. Were it not for the commonly observed segregation failures, a large portion of the waste generated could be recycled or recovered^(15,16)^.

It is worth noting that both recycling and recovery of medical devices present a number of disadvantages and implementation challenges^(17)^. Issues such as recyclability and commercial viability greatly impact the feasibility of these options. Thus, the focus of healthcare professionals should be, first and foremost, on reducing consumption.

Considering that the volume of surgeries worldwide has been increasing and that this trend is expected to continue^(18,19)^, it is imperative that actions be taken in Surgical Centers to mitigate the impact on the planet. To break this vicious cycle^(5)^ of negative environmental impact generated by healthcare services, mitigation measures must be implemented.

Studies have shown many possibilities for sustainable actions that can be implemented in Surgical Centers. Several possibilities can be implemented within the physical and material infrastructure itself and using the human resources already available in these sectors. Other proposals, however, require that measures for disease prevention and revisions to treatment choices be instituted, so that these are less invasive or interventionist^(5,20)^.

There are several proposed—and even already implemented—possibilities available in the scientific literature, such as eliminating the use of anesthetic gases with a high environmental impact, such as nitrous oxide; the conscientious use of oxygen in the PACU and during surgeries; installing motion sensors for lighting and air conditioning; the reuse of medical devices; the proper use of Personal Protective Equipment (PPE) and sanitizers; the revision of surgical kits and boxes; the replacement of disposable materials with reusable ones; well-managed inventories and consistent expiration dates for use in the CSSD; proper waste segregation; attention to the energy consumption of equipment in *standby* mode; etc.

It should be emphasized that for these actions to have an impact, institutions must invest in training and capacity building^(21)^. They must also identify potential environmentally-focused transformational leaders who aim to influence workers to engage in voluntary pro-environmental behavior^(22)^, as the involvement of local communities in decision-making processes is an important aspect of sustainable development.

Sustainable development is development that meets the needs of the present without compromising the ability of future generations to meet their own needs^(23)^. Surgical Centers Nurses can lead initiatives that synergistically integrate government, businesses, universities, civil society, and the natural ecological environment of these communities, taking ecological needs into account in the production of knowledge, innovation, and implementation of protocols, in what is known as the quintuple helix model^(24)^.

Taking intelligent and informed measures to promote equity in various settings, ensuring Planetary Health, is also a responsibility of Surgical Centers professionals. Therefore, contextualizing, developing solutions, and enabling the implementation of actions that align with the 17 Sustainable Development Goals (SDG) and their corresponding targets will help mitigate the negative impacts of care provided in the operating room on the health of the planet.

Environmentally sound, evidence-based measures aimed at conserving the planet’s natural resources and habitat, without threatening the safety of patients or professionals involved in care, must be taken. To this end, awareness, education, and a willingness to change are necessary to enable a virtuous cycle in perioperative healthcare and in Surgical Centers as a whole. Believing that changes must come solely from the government or commercial organizations outsources responsibility for Planetary Health, a responsibility that also lies with the individual and the health professional. It is up to all of us.

## References

[1] Keck F, Peller T, Alther R, Barouillet C, Blackman R, Capo E (2025). The global human impact on biodiversity.. Nature..

[2] Keil M, Frehse L, Hagemeister M, Knieß M, Lange O, Kronenberg T (2024). Carbon footprint of healthcare systems: a systematic review of evidence and methods.. BMJ Open..

[3] Rodríguez-Jiménez L, Romero-Martín M, Spruell T, Steley Z, Gómez-Salgado J (2023). The carbon footprint of healthcare settings: a systematic review.. J Adv Nurs..

[4] Braithwaite J, Smith CL, Leask E, Wijekulasuriya S, Brooke-Cowden K, Fisher G (2024). Strategies and tactics to reduce the impact of healthcare on climate change: systematic review.. BMJ.

[5] Brighton & Sussex Medical School. (2023). Centre for Sustainable Healthcare. UK Health Alliance on Climate Change. Green surgery: reducing the environmental impact of surgical care (v1.1) [Internet]..

[6] Karliner J, Slotterback S, Boyd R, Ashby B, Steele K (2019). Health care’s climate footprint: how the health sector contributes to the global climate crisis and opportunities for action. Healthcare without harm [Internet].. Brussels: HCWH.

[7] MacNeill AJ, Lillywhite R, Brown CJ (2017). The impact of surgery on global climate: a carbon footprinting study of operating theatres in three health systems.. Lancet Planet Health..

[8] Robinson PN, Surendran K, Lim SJ, Robinson M (2023). The carbon footprint of surgical operations: a systematic review update.. Ann R Coll Surg Engl..

[9] de’Angelis N, Conso C, Bianchi G, Rodríguez AGB, Marchegiani F, Carra MC (2024). Systematic review of carbon footprint of surgical procedures.. J Visc Surg.

[10] Rizan C, Steinbach I, Nicholson R, Lillywhite R, Reed M, Bhutta MF (2020). The carbon footprint of surgical operations: a systematic review.. Ann Surg..

[11] Jain N, LaBeaud D (2022). How should US Health Care Lead Global Change in Plastic Waste Disposal?. AMA J Ethics.

[12] Shoham MA, Baker NM, Peterson ME, Fox P (2022). The environmental impact of surgery: a systematic review.. Surgery..

[13] World Health Organization. (2018). Economics of the health implications of waste management in the context of a circular economy [Internet].. Geneve: WHO.

[14] World Health Organization. (2024). Fact sheets. Health-care waste [Internet].. Geneve: WHO.

[15] Amariglio A, Depaoli D (2021). Waste management in an Italian Hospital’s operating theatres: an observational study.. Am J Infect Control.

[16] Flynn FM, Martinsen SS, Øiseth F, Flo J, Leonardsen AL (2025). Sustainability in the operating room: a cross-sectional survey of nurse anaesthetists’ and operating room nurses’ views and practice.. BMC Nurs.

[17] Vergara R, Théate I, Boor P, Gordon IO, West J, Abdelmoula S (2025). Surgical pathology and sustainable development: international landscape and prospects.. J Clin Pathol..

[18] Triana L, Palacios RMH, Campilgio G, Liscano E (2024). Correction: trends in surgical and nonsurgical aesthetic procedures: a 14-Year Analysis of the International Society of Aesthetic Plastic Surgery-ISAPS.. Aesth Plast Surg.

[19] Weiser TG, Regenbogen SE, Thompson KD, Haynes AB, Lipsitz SR, Berry WR (2008). An estimation of the global volume of surgery: a modelling strategy based on available data.. Lancet..

[20] Kherad O, Peiffer-Smadja N, Karlafti L, Lember M, Aerde NV, Gunnarsson O (2020). The challenge of implementing Less is More medicine: a European perspective.. Eur J Intern Med.

[21] Felek BNE, Erkoç SK, Özçelik M, Yörükoğlu D (2025). Impact of waste segregation training on medical and recyclable waste in an operating theater a quasi experimental study.. Sci Rep.

[22] Nduneseokwu CK, Harder MK (2023). Developing environmental transformational leadership with training: leaders and subordinates environmental behaviour outcomes.. J Clean Prod.

[23] Brundtland GH (1987). Our common future: report of the world commission on environment and development. UN-Dokument A/42/427 [Internet].. United Nations.

[24] Rowan NJ (2024). Digital technologies to unlock safe and sustainable opportunities for medical device and healthcare sectors with a focus on the combined use of digital twin and extended reality applications: a review.. Sci Total Environ.

