# Supplementary material for: Sustainability: the role of the Surgical Center
Source: Rev Esc Enferm USP. 2026 Jun 1;60:e20260177. doi: 10.1590/1980-220X-REEUSP-2026-0177en (PMC13225252; doi:10.1590/1980-220X-REEUSP-2026-0177en)
Supplement: Translation

# Translation: Sustentabilidade: o papel do Bloco Operatório

**DOI:** 10.1590/1980-220X-REEUSP-2026-0177pt

**Authors:** Camila Quartim de Moraes Bruna

**Affiliations:** 1 Universidade de São Paulo, Escola de Enfermagem, Departamento de Enfermagem Médico-Cirúrgica, São Paulo, SP, Brasil.; 2 Universidade de São Paulo, Escola de Enfermagem, Comissão de Sustentabilidade, São Paulo, SP, Brasil.

## INTRODUÇÃO

O impacto gerado pelas ações humanas no planeta está bem estabelecido e muitas discussões são feitas relacionadas a ele^(1,2,3)^. Em contrapartida, menos atenção tem sido dada ao impacto dos serviços de saúde no planeta^(4)^. Reconhece-se que atualmente os serviços de saúde vivenciam um ciclo vicioso, no qual estes geram grandes emissões e impactos ao planeta, que afetam a saúde dos humanos, que por consequência, precisam usar mais os serviços de saúde, o que gera mais impacto ao planeta^(5)^.

Acredita-se hoje que se os serviços de saúde fossem um país, estariam na quinta colocação dentre os maiores emissores de gases de efeito estufa^(6)^. Dentre os setores hospitalares, o Bloco Operatório, composto por Centro Cirúrgico (CC), Recuperação Pós-Anestésica (RPA) e Centro de Material e Esterilização (CME), se destaca como grande gerador de pegada de carbono^(7)^. Considerando-se apenas a pegada de carbono dos CC, estima-se valores entre 28 kg a 505 kg de dióxido de carbono equivalente (CO_2_e) ^(8)^. Embora as quantidades de emissões variem muito dependendo de uma série de fatores, considera-se que um único procedimento cirúrgico emita de 4 a 814 kgCO_2_e^(9,10)^.

Esta importante pegada de carbono é advinda principalmente dos gases anestésicos e do consumo de energia^(7,10)^, mas a grande quantidade de resíduos gerados, principalmente provenientes de materiais descartáveis^(8)^, também tem grande impacto. Gera-se, apenas nos serviços de saúde dos Estados Unidos da América, cerca de 6 milhões de toneladas de resíduos por ano^(11)^. Além disso, um agravante é o fato de grande parte dos resíduos gerados no CC ser segregada incorretamente^(12)^.

O setor de saúde tem se tornado cada vez mais dependente de materiais de uso único, embora esses materiais reflitam um modelo de economia linear inerentemente insustentável, em que a gestão de resíduos cria um ônus significativo para a saúde pública em termos de poluentes ambientais^(13)^. Neste modelo os itens são produzidos, usados uma única vez e depois descartados.

Dentre o total de resíduos gerados na assistência à saúde, 85% é considerado resíduo comum, comparável aos resíduos domésticos, enquanto apenas 15% seria composto por material infeccioso, tóxico, carcinogênico, inflamável, corrosivo, reativo, explosivo ou radioativo^(14)^. Não fosse pelas falhas de segregação comumente observadas, grande parte dos resíduos gerados poderiam ser reciclados ou recuperados^(15,16)^.

Vale destacar que tanto reciclagem como recuperação de dispositivos médicos têm uma série de desvantagens e dificuldades de implementação^(17)^. Questões como reciclabilidade e interesse comercial impactam enormemente a viabilidade dessas possibilidades. Assim, o foco dos profissionais de saúde deve ser, primeiramente, reduzir o consumo.

Considerando-se que o volume de cirurgias no mundo todo vem aumentando e que há previsão de manutenção dessa tendência^(18,19)^, é imperativo que ações sejam tomadas nos Blocos Operatórios, a fim de mitigar o impacto causado ao planeta. Para que seja possível romper o ciclo vicioso^(5)^ do impacto ambiental negativo gerado pelos serviços de saúde, ações de mitigação precisam ser implementadas.

Estudos têm mostrado muitas possibilidades de ações sustentáveis possíveis de serem implementadas em Blocos Operatórios. Várias possibilidades podem ser implantadas na própria estrutura física e material e com os recursos humanos já existentes nestes setores. Já outras propostas requerem que ações na prevenção de doenças e revisões nas escolhas de tratamento sejam instituídas, para que estes sejam menos invasivos ou intervencionistas^(5,20)^.

Várias são as possibilidades propostas, e até já implementadas, disponíveis na literatura científica, como eliminar o uso de gases anestésicos com grande impacto ambiental, como o óxido nitroso; o uso consciencioso de oxigênio na RPA e durante as cirurgias; instalar sensores de presença para iluminação e ar condicionado; o reuso de dispositivos médicos; o uso adequado de Equipamentos de Proteção Individual e de saneantes; a revisão de kits e caixas cirúrgicas; a substituição de materiais descartáveis por reutilizáveis; inventários bem administrados e prazos limites para uso coerentes no CME; a segregação adequada dos resíduos; atenção ao gasto energéticos de equipamentos na função *stand by*; etc.

Ressalta-se que para que essas ações tenham impacto, as instituições devem investir em treinamentos e capacitações^(21)^. Bem como identificar possíveis lideranças transformacionais ambientalmente específicas, que visem influenciar trabalhadores a se envolverem em um comportamento voluntário pró-ambiental^(22)^, pois o envolvimento das comunidades locais nos processos de tomada de decisão é um importante aspecto do desenvolvimento sustentável.

O Desenvolvimento Sustentável é aquele que satisfaz as necessidades do presente, sem comprometer a capacidade das gerações futuras de suprir suas próprias necessidades^(23)^. Enfermeiros dos Blocos Operatórios podem estar à frente das iniciativas que integram de forma sinérgica governo, empresas, universidades, sociedade civil e o ambiente ecológico natural das comunidades, considerando necessidades ecológicas na produção de conhecimento, inovação e implementação de protocolos, no que é conhecido como modelo de hélice quíntupla^(24)^.

Tomar medidas inteligentes e informadas para promover a equidade em vários ambientes, garantindo a Saúde Planetária, é também função dos profissionais do Bloco Operatório. Assim, contextualizar, desenvolver soluções e possibilitar a implementação de ações que dialoguem com os 17 Objetivos de Desenvolvimento Sustentável (ODS), e suas metas correspondentes, permitirá mitigar os impactos negativos da assistência prestada no Bloco Operatório à saúde do planeta.

Medidas ecologicamente corretas, embasadas em evidências, que visem conservar os recursos naturais e o habitat do planeta, sem ameaçar a segurança do paciente ou profissionais envolvidos na assistência prestada, devem ser tomadas. Para tanto, é necessário sensibilização, conscientização e disposição para mudar, a fim de possibilitar um ciclo virtuoso na assistência à saúde perioperatória e nos Blocos Operatórios como um todo. Acreditar que as mudanças devem vir apenas do Estado ou organizações comerciais terceiriza a responsabilidade relacionada à Saúde Planetária, que também é do indivíduo, do profissional. É de todos nós.

# Translation: Sustentabilidad: el papel del Quirófano

**DOI:** 10.1590/1980-220X-REEUSP-2026-0177es

**Authors:** Camila Quartim de Moraes Bruna

**Affiliations:** 1 Universidade de São Paulo, Escola de Enfermagem, Departamento de Enfermagem Médico-Cirúrgica, São Paulo, SP, Brasil.; 2 Universidade de São Paulo, Escola de Enfermagem, Comissão de Sustentabilidade, São Paulo, SP, Brasil.

## INTRODUCCIÓN

El impacto que generan las acciones humanas en el planeta está ampliamente demostrado y se han realizado numerosos debates al respecto^(1,2,3)^. En cambio, se ha prestado menos atención al impacto de los servicios de salud en el planeta^(4)^. Se reconoce que, actualmente, los servicios de salud se encuentran en un círculo vicioso, en el que generan grandes emisiones e impactos en el planeta, lo que afecta la salud de las personas; estas, a su vez, necesitan recurrir más a los servicios de salud, lo que genera un mayor impacto en el planeta^(5)^.

Hoy en día se cree que si los servicios de salud fueran un país, ocuparían el quinto lugar entre los mayores emisores de gases de efecto invernadero^(6)^. Entre los sectores hospitalarios, el Quirófano, compuesto por las Salas de Operaciones (SO), la Recuperación Postanestésica (RPA) y el Centro de Materiales y Esterilización (CME), se destaca como un gran generador de huella de carbono^(7)^. Si se considera únicamente la huella de carbono de las SO, se estiman valores entre 28 kg y 505 kg de dióxido de carbono equivalente (CO_2_e)^(8)^. Aunque las cantidades de emisiones varían mucho dependiendo de una serie de factores, se considera que un solo procedimiento quirúrgico emite entre 4 y 814 kg de CO_2_e^(9,10)^.

Esta importante huella de carbono proviene principalmente de los gases anestésicos y del consumo de energía^(7,10)^, pero la gran cantidad de residuos generados, principalmente procedentes de materiales desechables^(8)^, también tiene un gran impacto. Solo en los servicios de salud de los Estados Unidos de América se generan alrededor de 6 millones de toneladas de residuos al año^(11)^. Además, un factor agravante es el hecho de que gran parte de los residuos generados en las SO se segrega incorrectamente^(12)^.

El sector de la salud se ha vuelto cada vez más dependiente de materiales de un solo uso, aunque estos materiales reflejan un modelo de economía lineal inherentemente insostenible, en el que la gestión de residuos crea una carga significativa para la salud pública en términos de contaminantes ambientales^(13)^. En este modelo, los artículos se producen, se usan una sola vez y luego se desechan.

Del total de residuos generados en la atención de la salud, el 85 % se considera residuo común, comparable a los residuos domésticos, mientras que solo el 15 % estaría compuesto por material infeccioso, tóxico, carcinógeno, inflamable, corrosivo, reactivo, explosivo o radiactivo^(14)^. De no ser por las fallas de segregación de desechos que se observan comúnmente, gran parte de los residuos generados podrían reciclarse o recuperarse^(15,16)^.

Cabe destacar que tanto el reciclaje como la recuperación de dispositivos médicos presentan una serie de desventajas y dificultades de implementación^(17)^. Cuestiones como la reciclabilidad y el interés comercial tienen un enorme impacto en la viabilidad de estas posibilidades. Por lo tanto, el enfoque de los profesionales de la salud debe ser, en primer lugar, reducir el consumo.

Teniendo en cuenta que el volumen de cirugías en todo el mundo viene aumentando y que se prevé que esta tendencia se mantenga^(18,19)^, es imperativo que se tomen medidas en los Quirófanos con el fin de mitigar el impacto causado al planeta. Para que sea posible romper el círculo vicioso^(5)^ del impacto ambiental negativo generado por los servicios de salud, es necesario implementar medidas de mitigación.

Los estudios han mostrado muchas posibilidades de acciones sostenibles que pueden implementarse en los Quirófanos. Varias de ellas pueden implementarse en la propia estructura física y material y con los recursos humanos ya existentes en estos sectores. Otras propuestas, en cambio, requieren que se establezcan medidas de prevención de enfermedades y revisiones en las opciones de tratamiento, para que estos sean menos invasivos o intervencionistas^(5,20)^.

Son varias las posibilidades propuestas, e incluso ya implementadas, disponibles en la literatura científica, como eliminar el uso de gases anestésicos con gran impacto ambiental, como el óxido nitroso; el uso consciente del oxígeno en la sala de recuperación postanestésica y durante las cirugías; instalar sensores de presencia para la iluminación y el aire acondicionado; la reutilización de dispositivos médicos; el uso adecuado de equipos de protección individual y de productos de higiene; la revisión de kits y maletines quirúrgicos; la sustitución de materiales desechables por reutilizables; inventarios bien administrados y plazos de uso coherentes en el CME; la segregación adecuada de los residuos; la atención al gasto energético de los equipos en modo *stand by*; etc.

Cabe destacar que, para que estas acciones tengan impacto, las instituciones deben invertir en capacitación y formación^(21)^. Así como identificar posibles líderes transformacionales específicos en materia ambiental, que busquen influir en los trabajadores para que se involucren en un comportamiento voluntario a favor del medio ambiente^(22)^, ya que la participación de las comunidades locales en los procesos de toma de decisiones es un aspecto importante del desarrollo sostenible.

El Desarrollo Sostenible es aquel que satisface las necesidades del presente sin comprometer la capacidad de las generaciones futuras para satisfacer sus propias necesidades^(23)^. Los enfermeros de los Quirófanos pueden estar al frente de iniciativas que integren de manera sinérgica al gobierno, las empresas, las universidades, la sociedad civil y el entorno ecológico natural de estas comunidades, considerando las necesidades ecológicas en la producción de conocimiento, la innovación y la implementación de protocolos, en lo que se conoce como modelo de hélice quíntuple^(24)^.

Tomar medidas inteligentes y informadas para promover la equidad en diversos entornos, garantizando la salud planetaria, es también una función de los profesionales del Quirófano. Así, contextualizar, desarrollar soluciones y hacer posible la implementación de acciones que dialoguen con los 17 Objetivos de Desarrollo Sostenible (ODS) y sus metas correspondientes permitirá mitigar los impactos negativos de la atención prestada en el Quirófano sobre la salud del planeta.

Se deben tomar medidas ecológicamente correctas, basadas en la evidencia, que busquen conservar los recursos naturales y el hábitat del planeta, sin amenazar la seguridad del paciente ni de los profesionales involucrados en la atención prestada. Para ello, es necesario la sensibilización, la concienciación y la disposición al cambio, con el fin de hacer posible un ciclo virtuoso en la atención de salud perioperatoria y en los quirófanos en su conjunto. Creer que los cambios deben provenir únicamente del Estado o de organizaciones comerciales, externaliza la responsabilidad relacionada con la salud planetaria, que también recae en el individuo, en el profesional. Es responsabilidad de todos nosotros.
